# Health Literacy and Clear Communication Best Practices for Telemedicine

**DOI:** 10.3928/24748307-20200924-01

**Published:** 2020-09-24

**Authors:** Cliff Coleman

Since the coronavirus 2019 (COVID-19) pandemic began unfolding in the United States in early 2020, health care professionals across the country have supported social distancing recommendations by offering telemedicine (ie, health care delivered to patients at a distance via telephone or two-way video) encounters in place of some in-person health care visits. (The broader category of telehealth, which includes a range of electronic and telecommunications approaches to communicating across the spectrum of health care stakeholders, is beyond the scope of this perspective.) Although communicating with patients by phone has long been a common practice for brief interactions, many clinicians are now conducting entire encounters via telemedicine.

About two-thirds of this author's recent academic medical center family medicine clinic encounters have been conducted via telemedicine. On a recent morning, for example, while working from home, I “saw” nine patients, scheduled for 20 minutes each: six via telephone, and three via secure two-way video. Issues addressed included chronic adrenal insufficiency (underactive adrenal glands), acute alcoholic hepatitis (liver inflammation due to alcohol use), new diagnoses of hypertension (high blood pressure) and plantar fasciitis (painful soft tissue injury of the foot), symptomatic atrial fibrillation (irregular heart beat rhythm), laboratory and radiograph results for a patient worried about lung cancer, poorly controlled type 2 diabetes, chronic low back pain, follow-up for pyelonephritis (bacterial infection of a kidney) in a man who tested positive for urinary tract chlamydia and gonorrhea (sexually transmitted infections), and medication assistance for smoking cessation in a person with schizophrenia who recently experienced a transient ischemic attack (temporary brain stroke).

In the 16 years since the publication of *Health Literacy: A Prescription to End Confusion* (Kindig et al., 2004) by the Institute of Medicine (now known as the National Academies of Science, Engineering and Medicine) we have made great strides in our understanding of the effects that low health literacy—defined as a person's ability to obtain, process, understand, and communicate basic health information and services needed to make health decisions (Somers & Mahadevan, 2010)—has on clinical outcomes (Berkman et al., 2011) for the 36% of U.S. adults who have limited health literacy skills at baseline (Kutner et al., 2006), and the rest of the population who is at risk for low health literacy any time they are sick, scared, sleep deprived, or hurt. We have also learned much about the clear communication best practices that health care professionals can use to help mitigate the impact of low health literacy (Coleman et al., 2013; Coleman et al. 2017). Available data suggest that many clear communication best practices were not in consistent widespread use prior to the COVID-19 crisis (Coleman et al., 2013; Coleman et al., 2017). The increasing use of telemedicine raises a variety of new issues related to health literacy and clear communication.

## Communicating via Telemedicine

Studies on the clinical effectiveness of telemedicine have had mixed results (Ekeland et al., 2010), and little is known about the effectiveness of communication during telemedicine encounters, with available studies showing mixed results here as well (Miller & Nelson, 2005). Interaction analysis has shown significant differences in communication behaviors between telemedicine and in-person patient-physician encounters (Agha et al., 2009), and there is reason to believe that significant communication differences occur between telephonic and two-way video interactions as well (Miller, 2002). Nonverbal communication is an important aspect of doctor-patient interactions, but little is known about its effects on patients' communication behaviors, including its ability to either amplify or counteract the meaning of spoken messages (Roter et al., 2006). Any effects of nonverbal body language are lost during telephone encounters and may be diminished during video encounters. For example, it may be difficult to detect patients' facial expressions or gestures indicating lack of understanding or disapproval, and patients may not be able to accurately gauge health care professionals' level of concern. Conversational continuers such as “I see” or “Go on” may be needed to replace an inquisitive look; turn-taking may occur less smoothly without full access to nonverbal cues or as a result of technological delays in transmission (Miller & Nelson, 2005); and it is not known how communicating via telemedicine affects clinicians' tendency to interrupt patients. Little is known about the effects on comprehension when health care personnel or patients speak with a significant accent. People who are hearing-impaired may be at a particular disadvantage during telemedicine encounters.

Some clear communication best practices will be more challenging via telemedicine. For example, the recommendation to speak slowly and clearly (Coleman et al., 2017) may depend on the quality of audio or internet connections and transmission delays (Miller & Nelson, 2005). In one small study, patients sought repetition more often during telemedicine encounters than during in-person encounters (Agah et al., 2009). Some clinicians draw pictures to help convey complex concepts related to anatomy or physiology, but this will not be possible via phone and may be challenging via video.

Other best practices, however, may be just as easily used via telemedicine. For example, adherence to a universal precautions approach to health communication (DeWalt et al., 2010), agenda-setting, using plain language, minimizing information overload, and using teach back (Coleman et al., 2017) all lend themselves to telemedicine encounters.

In some ways, telemedicine could even help improve communication. Being able to interact with health care team members remotely may help reduce situational anxiety associated with seeking services in a health care facility, which could help improve attention and, thus, retention and recall of information. In addition, it may be easier in some situations for a patient to have a family member listen in on the conversation when conducted via telemedicine to help provide a second set of ears; however, this raises potential ethical concerns related to privacy (see **Table 1** for suggestions). It remains to be seen what effects telemedicine may have on patients' trust and openness with health care personnel.

## Telemedicine, Health Literacy, and Health Disparities

Low health literacy disproportionately affects racial and ethnic minorities, people with lower socioeconomic status, people for whom English is not their first language, people with less education, and the elderly (Kutner et al, 2006). These groups also tend to experience high burdens of chronic disease as well as acute illness (including COVID-19), and persistent digital health disparities. For example, the 10% of U.S. citizens who do not have internet access is comprised mostly of people older than age 65 years, people who identify as African American or Hispanic, and people living in rural areas (Jackson et al., 2020). People and communities with lower income may also have less reliable access to telephone service, which is needed for telehealth encounters. These groups could experience worsening health disparities as the result of increased use of telemedicine. In contrast, some populations for whom transportation, work schedules, or caregiving demands have traditionally been barriers to accessing facility-based health services could benefit from increased access to care via telemedicine.

## Clear Communication Best Practices

The following clear communication best practices were developed by expert consensus for personnel working in all health professions for communicating with patients or caregivers in-person, telephonically, or in writing (Coleman et al., 2013; Coleman et al., 2017). Clear communication occurs when information is easy to understand and act on the first time it is heard (Pfizer, 2004). Although clear communication best practices have not been specifically determined for telemedicine, the following practices hold promise for optimizing high-quality communication during telephone and two-way video encounters.

### Follow a Health Literacy Universal Precautions Approach

Universal precautions for health communication assumes that all patients are at risk for miscommunication and misunderstanding, regardless of education, socioeconomic status, or literacy skills (DeWalt et al., 2010); recognizes that patients may go to great lengths to conceal their lack of understanding (Parikh et al., 1996); and acknowledges that health care professionals are poor at detecting when patients do not understand (Coleman, 2011). Health care personnel should use the same level of plain language and clear communication practices to lower barriers to understanding for all patients, whether interacting via telemedicine or in-person.

### Use Professional Medical Interpreters

About 22% of Americans speak a language other than English at home (U.S. Census Bureau, 2019). People with limited English proficiency are significantly more likely to have low health literacy (Kutner et al, 2006) and to experience health disparities and preventable medical errors. Use of untrained lay interpreters, such as family or friends, should be avoided because they frequently lack appropriate medical vocabulary; omit, add, or misinterpret information; and their presence may inhibit patients and health care personnel from discussing issues openly. The use of professional interpreters is associated with fewer communication errors, better comprehension, less unnecessary testing, lower risk of adverse events, better adherence to plans, shorter hospital stays, lower 30-day readmission rates, greater patient satisfaction, lower malpractice risk (Juckett & Unger, 2014), and is a top-rated clear communication practice (Coleman et al, 2017). Telephonic interpreter service providers and video-based conferencing platforms can generally establish a three-way connection between the patient, health care professional, and interpreter.

### Establish the Agenda at the Outset

Up-front agenda-setting (i.e., eliciting all of a person's concerns at the outset of an encounter) may be more important than ever during telephonic and two-way video encounters. Patients commonly have more than one concern but often do not mention their main concern first. Without some of the nonverbal cues needed to detect patients' emotional states, clinicians may need strong agenda-setting skills to help avoid last minute, so-called “doorknob” (or, in this case, “Before you hang up…”) questions that come up at what the clinician believed was the end of the encounter (Baker et al., 2005).

### Use Plain Language and Avoid Unnecessary or Undefined Jargon

The use of unnecessary or undefined medical jargon is a source of dissatisfaction for patients (McCarthy et al., 2012), yet even experienced clinicians frequently use jargon (Castro et al., 2007), which may impair patients' understanding of, and adherence to, their care plans. Health care professionals should strive to use plain “everyday” language with all patients (Coleman et al., 2017), and, when its use is necessary, clearly define all specialized medical terminology during telemedicine and in-person encounters.

### Avoid Information Overload

Lots of information is typically exchanged during medical encounters, but people's working memories have finite capacity for new information. About 50% of information is forgotten by patients immediately, and about one-half of what patients do recall following in-person encounters is incorrect (Kessels, 2003; McCarthy et al., 2012). In addition, patients may lack the clinical experience to determine which bits of information are more important than others. The effects of telephonic and two-way video communication characteristics on the quantity and quality of patient recall are not known. Clinicians are cautioned to avoid information overload by focusing on 1 to 3 “need-to-know” items per encounter if possible (Coleman et al., 2017).

### Provide Information Through Multiple and Preferred Channels

Most people learn best when information is presented through multiple communication channels, including spoken, written, graphic, and kinesthetic modalities. In an office setting, patients may have the advantage of hearing the clinician's voice, seeing their body language, and possibly being shown drawings, models, or videos to help make a concept clearer. To gauge a person's learning preferences, health care personnel may ask, “How do you like to learn about health information?” If the patient has access to an electronic patient portal, they may be able to receive written information, images, or links to online resources such as videos. During video encounters, clinicians may be able to use visual aids such as pictures, models, and a notepad or dry-erase board to spell out important medical terms with patients who are not visually impaired. With telephone encounters, the only available channel is voice. People who are hearing-impaired are at a disadvantage during in-person and telemedicine encounters but may benefit from the use of sign language interpretation during video encounters or the use of assistive technologies such as real-time captioning during telephone or video encounters (Spehar et al., 2016). People who are visually impaired may rely more heavily on auditory and kinesthetic channels during in-person encounters, which could pose particular challenges for clear communication during either telephone or two-way video encounters. Having health information provided in writing as a backup to spoken communication is an important tool for all patients and caregivers who read, because recall is so low for typical encounters (McCarthy et al, 2012).

### Summarize Near the End

Identify the key points and recommendations toward the end of the telemedicine encounter to re-orient patients to the most important information in a conversation. This can serve as a natural segue to eliciting any clarifying questions.

### Encourage Questions

All patients and caregivers should be encouraged to ask clarifying questions. Using a closed-ended approach, such as “Do you have any questions?” is felt to be ineffective because it frequently results in a “No” answer, even when the person may have questions. Indeed, in this author's experience, the use of a closed-ended format is associated with the use of discouraging nonverbal communication such as shaking one's head side-to-side or furrowing one's brow, signaling “No” or disapproval, which would be more of a concern during two-way video encounters than during telephone encounters. Instead, experts recommend using an open-ended format, such as “What questions do you have?” during all encounters, as this is felt to be more inviting and likely to elicit clarifying questions (Coleman et al., 2017).

### Use Teach-Back

The Teach-Back technique (asking patients to explain back key information in their own words) has been widely promoted as an important check on the clarity and quality of health communication, and has been identified as a top-rated communication practice (Coleman et al, 2017). Near the end of all telemedicine encounters health care personnel can say something like, “I want to make sure I've explained things clearly. In your own words, please explain the plan back to me.”

These clear communication best practices and a variety of other recommendations for patient-centered care during telemedicine encounters are listed in **Table 1**. During telemedicine encounters, patients should be encouraged to take notes if possible, and a written visit summary should be provided electronically or by mail if desired.

## Conclusion

The COVID-19 pandemic has accelerated the use of tele-medicine encounters. Because many medical encounters are relatively well-suited to this format, it seems likely that tele-medicine visits will remain with us even after the COVID-19 crisis passes. This presents specific communication challenges that health care workers should be prepared to address. Researchers should study the social dynamics of telemedicine interactions to empirically define and determine best practices for communicating with patients and caregivers in this way, with particular attention to the ways in which the use of telemedicine may affect health disparities.

## Figures and Tables

**Table 1:** Clear Communication Tips for Telemedicine Encounters

Preparing for encounters
When making appointments, confirm the patient's phone number and a back-up number if possible
Ask patients to make a list of concerns in advance
Use easy-to-read informed consent forms, if needed, for virtual visits (https://www.ahrq.gov/health-literacy/informed-consent-telemedicine.html)
Ask patients to gather data, such as vital signs or blood sugar readings, in advance
Ask patients to have prescription medication containers available for reference if needed
Make a backup plan in case the conversation gets cut off
Make sure phones or tablets are charged and have adequate prepaid minutes available
Have pen and paper ready to write a message in case the patient cannot hear you
Privacy
Both parties should find a quiet and private space to talk, if possible
Position yourself to avoid other people being visible in the background
Turn up volume on devices to a comfortable level
Identify yourself by name, role, and institution
Identify patients with two identifiers such as name and date of birth
Find out who, besides the patient, may be listening in, what their relationship is to the patient, and whether you have permission to discuss personal information
Tell patients if other health care personnel may be listening to your conversation
Use a headset to support privacy if needed
Develop and follow a policy for protecting privacy when leaving voice messages for patients
Hearing
Check that patients are hearing you adequately
Offer video sign language interpretation or captioning services to people who are hearing impaired
Clear communication
Use health literacy universal precautions for all encounters
Use professional medical interpreters
Establish the agenda at the outset
Use plain language; avoid unnecessary or undefined medical jargon
Avoid information overload; focus on 1 to 3 key massages
Repeat/summarize key information and recommendations
Use multiple teaching channels where possible, including visual aids, and written summaries
Elicit questions with an open-ended format, such as “What questions do you have?”
Use Teach-Back method to confirm clear communication and patient comprehension
Memory aids
Encourage patients and caregivers to have a pen and paper available for taking notes
If appropriate, encourage patients to have another person with them to provide a second set of ears
Arrange for patients to receive a written summary of the encounter
